# Anti‐PD‐1 monoclonal antibody‐resistant esophageal squamous cell carcinoma showing the abscopal effect: A case report with T‐cell receptor/B‐cell receptor repertoire analysis

**DOI:** 10.1002/cnr2.1832

**Published:** 2023-05-11

**Authors:** Yuka Takehara, Kosaku Mimura, Yoshiyuki Suzuki, Yohei Watanabe, Yuya Yoshimoto, Zenichiro Saze, Hisashi Sato, Tomoaki Tamaki, Koji Kono

**Affiliations:** ^1^ Department of Radiation Oncology Fukushima Medical University School of Medicine Fukushima Japan; ^2^ Department of Gastrointestinal Tract Surgery Fukushima Medical University School of Medicine Fukushima Japan; ^3^ Department of Blood Transfusion and Transplantation Immunology Fukushima Medical University School of Medicine Fukushima Japan

**Keywords:** abscopal effect, anti‐PD‐1 monoclonal antibody, B‐cell receptor repertoire, esophageal squamous cell carcinoma, radiation therapy

## Abstract

**Background:**

Several clinical trials of nivolumab have reported good results, including those in patients with advanced esophageal squamous cell carcinoma. However, the response rate of this drug remains poor. Notably, a rare phenomenon called abscopal effect refers to the regression of irradiated and nonirradiated distant tumors after local radiotherapy. Although the mechanism of this effect remains unclear, the antitumor immunity induced by radiotherapy is considered to be the most important factor.

**Case:**

A 66‐year‐old man with recurrent nivolumab‐resistant esophageal squamous cell carcinoma along with left‐side cervical and abdominal para‐aortic lymph node metastases was treated with a 40 Gy (10 fractions) dose of radiotherapy to the left‐side cervical lymph node metastasis as a palliative treatment, which caused neck pain. In addition, nivolumab administration was resumed the day after completion of radiotherapy. Three months after radiotherapy, the irradiated lesion on the left neck had regressed to a scar‐like lesion. Furthermore, the previously progressive abdominal para‐aortic lymph nodes outside the irradiation area shrank (abscopal effect). T‐cell receptor and B‐cell receptor (TCR/BCR) repertoire analyses before and after radiotherapy revealed that radiotherapy led to changes in the TCR/BCR repertoire.

**Conclusion:**

Changes in the TCR/BCR repertoire may be a part of the mechanism underlying the abscopal effect. The findings of the present case suggest that the combination of immune checkpoint inhibitors and radiotherapy is a promising treatment approach even for patients with immune checkpoint inhibitor‐resistant cancer.

## 1 INTRODUCTION

Although esophageal cancer is recognized as one of most fatal cancers worldwide,^1^ drugs proven to be effective against this cancer are fewer than those that are effective against cancers at other sites. In the United States and Europe, adenocarcinoma is the most common histological type. In contrast, squamous cell carcinoma is the main histological type in Asia, and the combination of cisplatin and 5‐fluorouracil has been used as a key approach in Japan.^2^

With the clinical success of immune checkpoint inhibitors (ICIs), such as anti‐PD‐1^3^ and anti‐CTLA‐4 antibodies,^4^ tumor immunity has attracted much attention in cancer treatment. In the 2000s, it became clear that immunity against tumors plays an important role in not only suppressing cancer development but also cell‐killing anticancer therapies, such as radiotherapy (RT) and chemotherapy.^5^

The ATTRACTION‐3 study is a phase III clinical trial with unresectable advanced or recurrent esophageal cancer that is refractory or intolerant to combination chemotherapy.^6^ Nivolumab use led to a significant increase in survival compared with chemotherapy (taxane). Thus, it was approved as a second‐line treatment for unresectable advanced, recurrent, or metastatic esophageal squamous cell carcinoma (ESCC) by the Food and Drug Administration and for unresectable advanced, recurrent, or metastatic esophageal cancers by the Pharmaceuticals and Medical Devices Agency, Japan, in 2020. However, the response rate was only 19%. The combination of nivolumab with other therapies to enhance its efficacy is being actively studied worldwide. Recently, the results of the CheckMate 648 Trial, a phase III clinical trial involving nivolumab combination therapy as a first‐line treatment in advanced ESCC were reported.^7^ Nivolumab plus chemotherapy and nivolumab plus ipilimumab resulted in significantly longer overall survival than chemotherapy alone, with no new safety signals identified. However, the median overall survival period was 13.7–15.4 months, and further improvement in the treatment protocol is still needed.

A rare phenomenon called the abscopal effect refers to the regression of not only the irradiated tumor but also non‐irradiated distant tumors after local RT. The mechanism is not completely clear, but it is thought that the activation of anti‐tumor immunity induced by RT is the main factor.^8^, ^9^ According to our previous report, tumor antigen‐specific cytotoxic T lymphocytes were induced by chemoradiotherapy in 38% of the patients with advanced ESCC.^9^ Recently, the abscopal effect was reported in patients treated with a combination of ICIs and RT (ICI + RT).^10^, ^11^ Postow et al.^10^ reported a melanoma patient treated with ipilimumab + RT showing an abscopal effect and changes in humoral immune responses, NY‐ESO‐1 titers, during abscopal effect. Although this effect is very rare in cases of esophageal cancer, some recent reports indicated the role of this effect in such cases.^12^, ^13^ In particular, Postow et al.^10^ reported the case of a melanoma patient with ipilimumab (ICI)‐resistant tumor treated with RT and ICI who exhibited an abscopal effect. Therefore, even if the tumor is resistant to ICI, it is worth trying to add RT to ICI.

Here, we report a patient with anti‐PD‐1 monoclonal antibody (mAb)‐resistant ESCC showing the abscopal effect (systemic response), and present the findings of T‐cell receptor (TCR) and B‐cell receptor (BCR) repertoire analysis before and after RT.

## 2 CASE PRESENTATION

**FIGURE 1:**
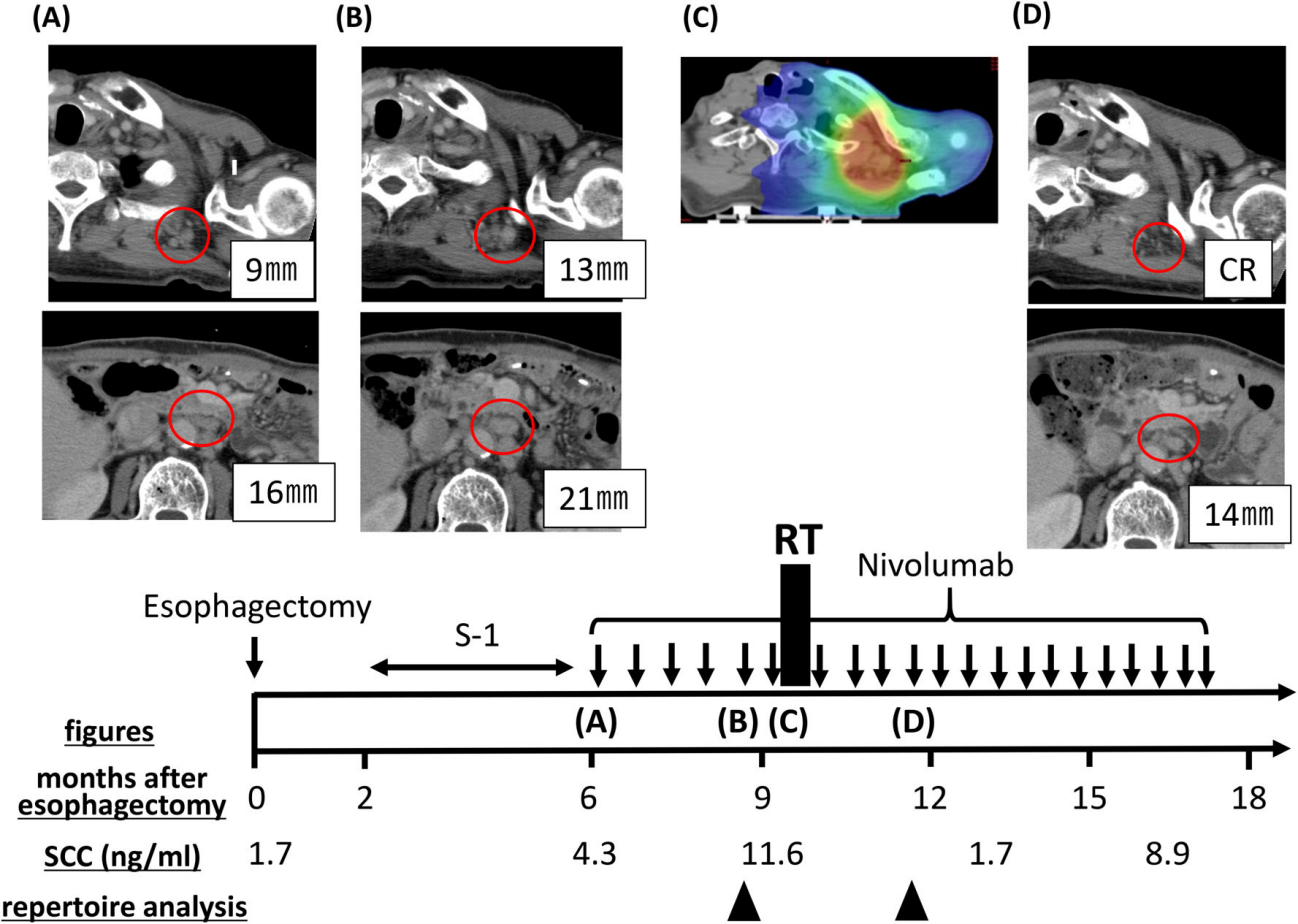
Results of diagnostic and radiotherapy simulation imaging throughout the disease course. Axial computed tomography (CT) images are shown, corresponding to the timeline showing therapy. Red circles indicate left‐neck lymph node metastasis and abdominal para‐aortic lymph node metastasis. (A) Representative figures before treatment with nivolumab. (B) Figures showing enlargement of the left‐neck lymph node and abdominal para‐aortic lymph node metastases. (C) Figure showing the CT simulation image for radiotherapy planning. The isodose paint represents total doses of >40 Gy (red), 20–36 Gy (green), and 12–20 Gy (blue). (D) Figures showing findings 3 months after radiotherapy. The irradiated left‐neck lymph node metastasis regressed to a scar‐like appearance. Furthermore, disease response outside of the radiation field was seen.

A 66‐year‐old man was diagnosed with advanced ESCC (Mt‐Lt, cT3N2M1, stage IVb). Histopathological examination of the endoscopic biopsy specimen of the patient revealed squamous cell carcinoma. Moreover, a computed tomography (CT) scan revealed thick esophageal wall and left‐side neck and mediastinum lymphadenopathy. Six weeks after the neoadjuvant chemotherapy (1 × 5‐fluorouracil + cisplatin and 1 × docetaxel + 5‐fluorouracil + cisplatin), he underwent esophagostomy with regional lymph node dissection at Fukushima Medical University Hospital in April 2020. Histopathological examination of the resected specimen revealed that the TNM classification of the tumor was ypT2 ypN4, and the histological efficacy of chemoradiotherapy was grade 1a. Further, the patient was discharged from the hospital 46 days after the surgery without any complications. Tegafur/gimeracil/oteracil (S‐1) was started as postoperative adjuvant chemotherapy from 2 months after the esophagostomy. Six months after esophagostomy, left‐side cervical and abdominal para‐aortic lymph node metastasis was detected on CT (shown in Figure 1A). Then, S‐1 was discontinued and nivolumab 240 mg per body was administered as second‐line treatment 6 times every 2 weeks. Two months later, a follow‐up CT scan revealed that all metastatic lymph nodes progressed, especially the left cervical lymph node (shown in Figure 1B), and serum squamous cell carcinoma (SCC) antigen levels increased to 11.6 ng/mL. In other words, these tumors were nivolumab resistant. To treat left‐side neck pain caused by the progressed metastatic lymph nodes, a total of 40 Gy (10 fractions) of RT was administered as a palliative treatment through the intensity‐modulated image‐guided technique (shown in Figure 1C), and nivolumab was continuously administered. Left‐side neck pain mostly disappeared at the end of RT. A CT scan at 3 months after RT showed that the irradiated lesion in the left neck had regressed to a scar‐like appearance. Notably, the abdominal para‐aortic lymph nodes outside the irradiation area, which previously tended to be progressive, also shrank (shown in Figure 1D), and serum SCC antigen levels decreased to 1.7 ng/mL.

**FIGURE 2:**
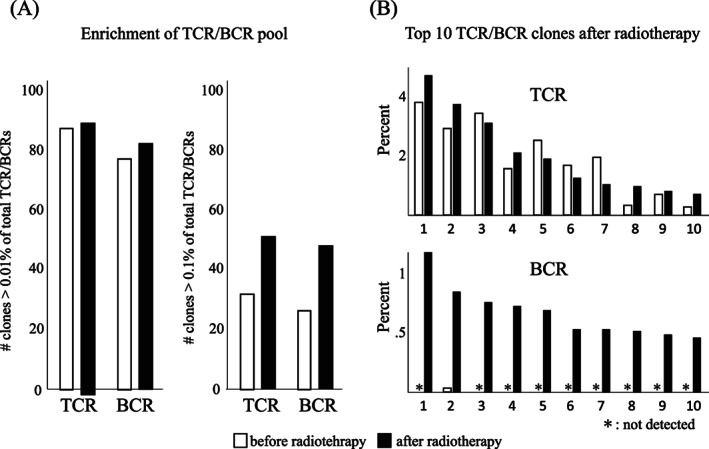
(A) Enrichment of the T‐cell receptor (TCR)/B‐cell receptor (BCR) pool in clones. There are slight increases in the percentage of unique clones accounting for >0.01% of total TCRs/BCRs before and after radiotherapy, and large increases in that of unique clones accounting for >0.1% of total TCRs/BCRs. (B) Top 10 TCR/BCR clones after radiotherapy. In the TCR repertoire, the top 10 clones are mostly similar to that before radiotherapy, but there are some changes (the percentages of the 1st, 2nd, and 4th clones increased more than 0.5%). In the BCR repertoire, all the top 10 clones before radiotherapy are replaced after radiotherapy. Furthermore, 9 of the 10 clones are newly detected after radiotherapy. □: before radiotherapy; ■: after radiotherapy; *: not detected.

The TCR and BCR repertoire analysis was performed by Repertoire Genesis Inc. (Osaka, Japan). Briefly, cDNA was synthesized from total RNA of peripheral blood mononuclear cells of the patient. Moreover, polymerase chain reaction was performed for TCR‐beta and BCR‐IgG, and unbiased next generation sequencing was performed for the analysis. Further, TCR/BCR sequences were assigned based on their similarity with the reference sequences from the international ImMunoGeneTics information system® (IMGT) database (http://www.imgt.org) using the repertoire analysis software (Repertoire Genesis, Inc.), and the number of unique clones was counted.^14^, ^15^ The diversity of the repertoires was described in terms of the values for Shannon–Weaver index *H*′, Inv. Simpson's index 1/*λ*, Pielou's evenness, and DE50. The diversity indices of the TCR and BCR repertoire analysis before and after RT are shown in Table 1. All diversity indices in both TCR and BCR repertoires decreased after RT. The numbers of unique clones, accounting for >0.01% and >0.1% of the total TCRs/BCRs before and after RT, are shown in Figure 2A, and the comparison of top 10 clones of TCRs/BCRs between before and after RT is shown in Figure 2B. There were slight increases in the percentage of unique clones accounting for >0.01% of total TCRs/BCRs before and after RT and large increases in that of unique clones accounting for >0.1% of total TCRs/BCRs before and after RT. The top 10 clones were mostly similar in the TCR repertoire (8 of the top 10 clones before RT were still in the top 10 clones after RT), but there were some changes (the percentages of the 1st, 2nd, and 4th clones increased more than 0.5%). In the BCR repertoire, all the top 10 clones before RT were replaced after RT. Furthermore, 9 of the 10 clones were newly detected after RT.

**TABLE 1:** (A) Diversity indexes of the T‐cell receptor repertoire analysis before and after radiotherapy. (B) Diversity indexes of the B‐cell receptor repertoire analysis before and after radiotherapy.

Index	Before	After
(A)		
Shannon‐Weaver index *H*′	7.36	6.86
Inverse Simpson's index 1/*λ*	174	148
Pielou's evenness	0.780	0.711
Diversity evenness 50	0.031	0.012
(B)		
Shannon‐Weaver index *H*′	8.16	7.61
Inverse Simpson's index 1/*λ*	1030	689
Pielou's evenness	0.812	0.774
Diversity evenness 50	0.023	0.015

Subsequently, the irradiated tumors exhibited complete response (for 20 months until his death), and the patient's condition progressed well. However, the abdominal para‐aortic lymph nodes re‐progressed and the serum SCC antigen levels increased again to 8.9 ng/mL 7 months after RT; thus, a total of 40 Gy (10 fractions) of RT was administered to the lymph nodes; these irradiated metastatic lymph nodes showed complete response until his death. Nivolumab was stopped due to nivolumab‐related skin rash (out of the irradiated area) at 10 months after the first RT (3 months after the second RT). At 11 months after the first RT, new lymph node metastases (inguinal and axillary) were detected. However, best supportive care was performed rather than cancer treatment owing to the patient's poor general condition, and the patient died due to ESCC 20 months after the first RT (i.e., 29 months after esophagostomy). The adverse events were as follows: grade 1 oral mucositis, dry skin, pruritus, and fatigue. However, no grade 2 or higher adverse events were observed while using RT along with ICI until the patient's death. Moreover, all adverse events were associated with nivolumab and not with RT.

## 3 DISCUSSION

We report the case of a patient with ESCC who was treated with RT and nivolumab as palliative local treatment after disease progression; moreover, he exhibited the abscopal effect during nivolumab treatment. TCR and BCR were analyzed before and after RT, and the results indicated changes in TCR and BCR repertoires after RT. Thus, we hypothesized that these immunological responses were a part of the mechanism of abscopal effect.

Chen and Mellman^5^ reported that RT plays a role in antigen spread, which leads to T‐cell immune activation in the cancer immunity cycle. Moreover, according to the review by Sharabi et al.,^8^ the roles of RT in cancer immunology are as follows: (1) radiation induces changes to the tumor cell immunophenotype, (2) radiation enhances cross‐presentation of tumor antigens, and (3) radiation combined with an ICI increases tumor cell susceptibility to immune‐mediated cell death. We have reported that RT‐induced cancer/testis antigen‐specific cytotoxic T‐cell activation in 38% of ESCC patients treated with chemo‐RT, and multi‐antigen‐specific T‐cell responses were observed in some patients.^9^

Already, the concept of ICI + RT (so‐called immunoradiotherapy) has been clinically proven in non‐small‐cell lung cancer (NSCLC).^10^, ^11^ The anti‐PD‐L1 antibody durvalumab administered every 2 weeks for 12 months after radical chemo‐RT for stage 3 NSCLC (PACIFIC trial) improved the progression‐free survival rate by about 20% at 1 year^16^ and the overall survival rate by about 15% at 4 years.^17^ This ICI + RT regimen has already become the standard therapy for stage 3 NSCLC. At present, many clinical trials of ICI + RT are underway for cancers at other sites, including esophageal cancer.^18^

Regarding the adverse effects of ICI + RT, severe (grade 3 or higher) adverse effects were not increased in the PACIFIC trial. Furthermore, Sha et al.^19^ performed a systemic review and meta‐analysis, and reported comparable grade 3–4 toxicity using ICI + RT compared with ICI alone in CNS melanoma, NSCLC, and prostate cancer, and they concluded that ICI + RT was safe. In the present case, the patient presented with the following adverse events: grade 1 oral mucositis, dry skin, pruritus, and fatigue. In contrast, no grade 2 or higher adverse events were observed while using the combination of RT and ICI until his death.

A change in the TCR/BCR repertoire after treatment is proof of a treatment‐induced immunoresponse. Changes in the TCR and BCR repertoires by treatment have already been reported.^20^, ^21^ Moreover, changes in the TCR repertoire by RT and chemo‐RT have been reported in myeloma,^20^ and head and neck cancer.^21^ The usefulness of analyzing the TCR repertoire to detect the efficacy of anti‐PD‐1 antibody (clonality [1 − Pielou's evenness]^22^ and frequency of the top 30 most frequent clonotypes^23^) and the BCR repertoire to detect the efficacy of autologous cellular immunotherapy (clonality^24^) has been reported. Someya et al.^25^ reported that dynamic changes in TCR could have a prognostic significance for non‐small‐cell lung cancer treated with ICI + (chemo)RT. Analysis of the TCR and BCR repertoires may facilitate the dissection and understanding of the immune response in human cancer patients and may be useful as a biomarker of RT or ICI + RT. In our patient, the BCR repertoire ranking greatly changed with RT and the TCR repertoire also changed. This means that all diversity indices for both the TCR and BCR repertoires decreased after RT. These changes in the TCR and BCR repertoires may be associated with RT‐induced tumor‐specific anti‐tumor immunity, resulting in the abscopal effect in the patient with nivolumab‐resistance. In other words, it is possible that further antigen spread by RT may retrieve the effect of nivolumab in patients with nivolumab‐resistance. Since each metastatic tumor is not the same (homogenous), RT to another metastatic tumor may induce new or more immune responses.^26^

In conclusion, we experienced the case of a patient who was treated with palliative local RT + nivolumab after disease progression during nivolumab treatment indicated the abscopal effect. Changes in the TCR and BCR repertoires were observed after RT, and these were assumed to be a part of the mechanism of the abscopal effect. The findings in this patient suggest that ICI + RT can be a promising treatment approach, even for patients with ICI‐resistant cancer.

## AUTHOR CONTRIBUTIONS

**Yuka Takehara:** Data curation (equal); resources (equal); visualization (equal); writing – original draft (lead); writing – review and editing (equal). **Yoshiyuki Suzuki:** Conceptualization (equal); data curation (equal); funding acquisition (lead); supervision (equal); visualization (equal); writing – original draft (supporting); writing – review and editing (equal). **Kousaku Mimura:** Conceptualization (equal); data curation (equal); resources (equal); writing – review and editing (equal). **Yuya Yoshimoto:** Resources (equal); writing – review and editing (equal). **Yohei Watanabe:** Resources (equal); writing – review and editing (equal). **Zenichiro Saze:** Resources (equal); writing – review and editing (equal). **Hisashi Sato:** Resources (equal); writing – review and editing (equal). **Tomoaki Tamaki:** Resources (equal); writing – review and editing (equal). **Koji Kono:** Supervision (equal); writing – review and editing (equal).

## CONFLICT OF INTEREST STATEMENT

The authors have stated explicitly that there are no conflicts of interest in connection with this article.

## ETHICS STATEMENT

*Patient consent*: The authors declare that they have obtained written informed consent from the patient.

## Data Availability

Data available on request due to privacy/ethical restrictions.
